# Acceptability of Community-Based Tuberculosis Preventive Treatment for People Living with HIV in Zimbabwe

**DOI:** 10.3390/healthcare10010116

**Published:** 2022-01-07

**Authors:** Martin K. Msukwa, Munyaradzi P. Mapingure, Jennifer M. Zech, Tsitsi B. Masvawure, Joanne E. Mantell, Godfrey Musuka, Tsitsi Apollo, Rodrigo Boccanera, Innocent Chingombe, Clorata Gwanzura, Andrea A. Howard, Miriam Rabkin

**Affiliations:** 1 ICAP, Columbia University, Centurion, Pretoria 0157, South Africa; 2 ICAP, Columbia University, Harare, Zimbabwe; mpm2189@cumc.columbia.edu (M.P.M.); gm2660@cumc.columbia.edu (G.M.); ic2421@cumc.columbia.edu (I.C.); 3 ICAP, Columbia University, New York, NY 10032, USA; jz2973@cumc.columbia.edu (J.M.Z.); aah2138@cumc.columbia.edu (A.A.H.); mr84@cumc.columbia.edu (M.R.); 4 Center for Interdisciplinary Studies, Health Studies Program, College of the Holy Cross, Worcester, MA 01610, USA; tmasvaure@yahoo.com; 5 HIV Center for Clinical and Behavioral Studies, Gender, Sexuality and Health Area, New York State Psychiatric Institute and Department of Psychiatry, Columbia University Irving Medical Center, New York, NY 10032, USA; jem57@cumc.columbia.edu; 6 Ministry of Health and Child Care, AIDS and TB Unit, Harare, Zimbabwe; tsitsiapollo2@gmail.com (T.A.); cloratag@gmail.com (C.G.); 7 Health Resources and Services Administration (HRSA) Office of Global Health, Rockville, MD 20857, USA; Rboccanera@hrsa.gov; 8 Department of Epidemiology, Mailman School of Public Health, Columbia University, New York, NY 10032, USA

**Keywords:** HIV, TB prevention, TPT, differentiated service delivery, Zimbabwe, integration

## Abstract

As Zimbabwe expands tuberculosis preventive treatment (TPT) for people living with HIV (PLHIV), the Ministry of Health and Child Care is considering making TPT more accessible to PLHIV via less-intensive differentiated service delivery models such as Community ART Refill Groups (CARGs). We designed a study to assess the feasibility and acceptability of integrating TPT into CARGs among key stakeholders, including CARG members, in Zimbabwe. We conducted 45 key informant interviews (KII) with policy makers, implementers, and CARG leaders; 16 focus group discussions (FGD) with 136 PLHIV in CARGs; and structured observations of 8 CARG meetings. KII and FGD were conducted in English and Shona. CARG observations were conducted using a structured checklist and time-motion data capture. Ninety six percent of participants supported TPT integration into CARGs and preferred multi-month TPT dispensing aligned with ART dispensing schedules. Participants noted that the existing CARG support systems could be used for TB symptom screening and TPT adherence monitoring/support. Other perceived advantages included convenience for PLHIV and decreased health facility provider workloads. Participants expressed concerns about possible medication stockouts and limited knowledge about TPT among CARG leaders but were confident that CARGs could effectively provide community-based TPT education, adherence monitoring/support, and TB symptom screening provided that CARG leaders received appropriate training and supervision. These results are consistent with findings from pilot projects in other African countries that are scaling up both differentiated service delivery for HIV and TPT and suggest that designing contextually appropriate approaches to integrating TPT into less-intensive HIV treatment models is an effective way to reach people who are established on ART but who may have missed out on access to TPT.

## 1. Introduction

Tuberculosis (TB) is the leading cause of death for people living with HIV (PLHIV), making the scale up of both antiretroviral therapy (ART) and TB preventive therapy (TPT) an important global priority. Opportunities to integrate TB screening, case finding, and prevention into HIV service delivery programs occur at multiple points, including at the time of HIV testing, at ART initiation, and during treatment.

As HIV treatment approaches diversify and become more person-centered and as differentiated service delivery (DSD) models are taken to scale, early evidence suggests that the integration of TPT into less-intensive differentiated ART models is a promising strategy [1]. A pilot project in Zambia, for example, integrated TPT into that country’s Fast Track treatment model, providing multi-month dispensing of both ART and TPT to adult PLHIV established on treatment at one health facility; preliminary results suggest this may have increased the uptake of TPT [1,2]. In South Africa, Doctors Without Borders and the City of Cape Town piloted the integration of TPT services into the ART Club model, and the success of this strategy led to further scale-up in the Western Cape Province [3].

As it is globally, TB is a leading cause of death for PLHIV in Zimbabwe, which had an adult HIV prevalence of 12.9%, a TB incidence amongst PLHIV of 193 per 100,000 people, and 5900 PLHIV deaths due to TB in 2019 [4,5]. In response, Zimbabwe’s Ministry of Health and Childcare (MoHCC) is scaling up both ART and TPT for PLHIV. More than 85% of PLHIV are now on ART [3], and the percentage of newly enrolled ART patients receiving TPT increased from 11% in 2017 [4] to 89% in 2019 [6].

People who have been enrolled in HIV care for years do not always have the same TPT access as PLHIV who are newly initiating ART, and extending TPT catch up services to this cohort is now a priority. Importantly, many long-term ART users are enrolled in less-intensive DSD models. By December 2020, 39% of Zimbabwe’s 1.1 million PLHIV on ART were enrolled in DSD, with 12% (132,000 people) enrolled in Community ART Refill Groups (CARGs) [7].

**Figure 1:**
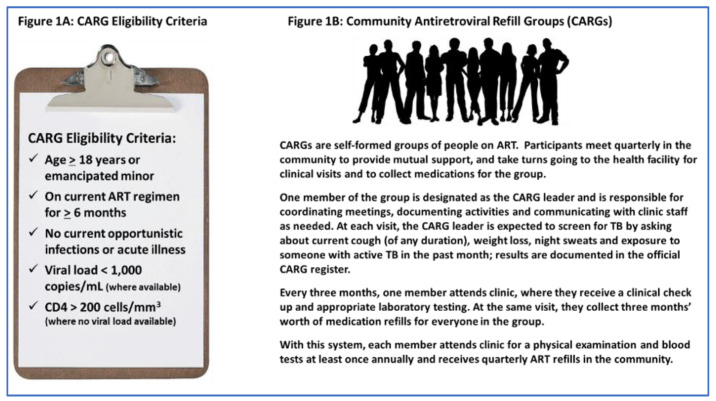
Community ART Refill Groups (CARGs) in Zimbabwe.

Zimbabwe’s CARG model is an opt-in peer-led community-based group treatment model. CARGs typically include 6–12 people (Figure 1A); they meet quarterly in the community to distribute ART, monitor adherence, screen for symptoms of illness including TB, and provide mutual support (Figure 1B). Each CARG has a peer leader, who is also on ART, who coordinates meetings, documents activities, and communicates with health facility (HF) staff.

While Zimbabwe’s national guidelines do not prioritize TPT for people in DSD models, interest in this approach is increasing [8,9,10,11]. In response, ICAP at Columbia University and MoHCC designed a study to explore the acceptability and perceived feasibility of integrating community-based TPT into CARGs.

## 2. Materials and Methods

### 2.1. Study Setting, Participant Recruitment, and Sample Size

Four urban and three rural public-sector HF with ≥ 1000 adults on ART and ≥ 150 people in CARGs were purposively selected to represent diverse settings, in consultation with the MoHCC and with the Zimbabwe National Network of People Living with HIV (ZNNP+).

We conducted 45 key informant interviews (KIIs), 16 focus group discussions (FGDs), and 8 CARG observations between May and September 2019. Data collection strategies, samples, and illustrative domains of inquiry are detailed in Table 1.

Twenty CARG leaders and 25 policy makers, funders, and implementers were identified via purposive sampling and were invited to participate in KIIs. FGDs included 136 purposively selected CARG members (6–12 participants per FGD, mean of 8). Half of the FGDs (n = 8) included members who had received TPT prior to participating in the study, and the other half had not. Clinicians and community linkage facilitators at the study sites identified a convenience sample of adults who were currently enrolled in CARGs and referred interested clients to the study team. The team met with potential FGD participants to explain the study, invited them to participate, and obtained informed consent.

A convenience sample of eight CARGs was selected for the structured observations. CARG leaders and CARG members were compensated for the time spent in the KIIs and FGDs with small non-monetary items valued at no more than USD 5.

### 2.2. Data Collection

Data collection took place between March 2019 and September 2019.

#### 2.2.1. Key Informant Interviews

Semi-structured in-person interviews were conducted in English and/or Shona (the local language spoken most frequently at the study sites). Interviews were audio-recorded, transcribed, translated into English as needed, and reviewed for accuracy and completeness by bilingual research staff before analysis. The SurveyCTO mobile data collection platform (Dobility Inc., Cambridge, MA, United States) was used to capture close-ended questions with a tablet-based interviewer-assisted form. Data were uploaded to SurveyCTO, with internal data quality checks for valid entries, skip patterns, range checks, and missing values.

#### 2.2.2. Focus Group Discussions

FGDs were stratified by their HF setting (urban/rural) and the participants’ experience with TPT (yes/no). They were conducted in Shona, audio-recorded, transcribed, and translated into English. A bilingual researcher validated each transcript for completeness and accuracy prior to coding.

#### 2.2.3. CARG Observations

The study staff used a checklist to observe CARG meetings and to document activities. SurveyCTO was used to create a tablet-based interviewer-assisted form, and data were uploaded to the SurveyCTO system with internal data quality checks.

We also asked the KII and FGD participants to characterize their response to three hypothetical TPT delivery models (Table 2) to explore their preferences, priorities, and concerns. Using a five-point scale that ranged from “strongly disagree” to “strongly agree”, we asked the KII and FGD participants if they agreed whether a specific model would be acceptable, feasible, and effective.

### 2.3. Data Analysis

The KIIs and FGDs were analyzed using the Dedoose web application (version 8.1.8, SocioCultural Research Consultants, LLC Los Angeles, CA, USA).

A team of five researchers coded transcripts by question and key themes; code reports were then synthesized into key findings. Quantitative data were analysed using SAS statistical software package (version 9.4, SAS Institute Inc., Cary, NC, USA).

## 3. Results

### 3.1. Participant Characteristics

The KII participants included 25 policy and program stakeholders: 11 MoHCC staff, 7 implementing partners, 3 ZNNP+ staff, 3 donors, and 1 academic partner. They had worked at their respective institutions for a median of 4 years (range 2–5); 40% were female. Twenty CARG leaders also participated in KIIs: 55% were female, all had been CARG leaders for at least 1 year, and 45% had been CARG members for at least 3 years.

Table 3 describes the 136 CARG members who participated in the FGDs. Their median age was 46 years (range 20–66; IQR 42–54), 68% were female, and their median time on ART was 8 years (range 1–19; IQR 6–11). Nearly half (49%) had at least one other household member in their CARG.

Sixty-four CARG members were observed during the eight CARG observations: 73% were female, their median time on ART was 8 years (range 4–18; IQR 6–12), all had been in a CARG for at least 1 year, and 30% had been CARG members for over 2 years.

### 3.2. Perceived Benefits of Providing TPT through CARGS

There was consensus across the participant groups that community-based TPT would benefit both CARG members and health care workers (HCW). Participants (which refers to all groups unless otherwise specified) highlighted leveraging existing CARG structures, decongesting HF, and offering convenience for CARG members as key advantages.

Use of an existing structure: Most of the participants in all groups favored providing TPT via CARGs, mentioning peer support, monitoring and supporting ART adherence, and providing education and psychosocial support to members as services that could easily be extended to TPT.

*...it’s a good strategy on the issue of adherence and monitoring each other. The strategy we are using to monitor each other on CARGs is the same that we [would] use to monitor each other on these tablets (i.e., TPT).*

- FGD CARG member participant (no TPT experience)

*I think we can also tap [into] and write on the benefits that have been shown to come from CARGs regarding ART retention and care…*

- Policy/program-level key informant

The CARG leaders expressed confidence in their ability to successfully manage TPT and did not think that integrating TPT into CARGs would overburden them or substantially change their medication collection schedules or the frequency of member monitoring.

*It won’t be much [work] because when we collect ARVs we can also collect TPT, so we will be collecting both and when we go for our review…after 3 months we will be taking care of both sides at the same time, so it won’t be much of a problem.*

- CARG leader key informant

Participants who had previously taken TPT highlighted the important role that CARG members could play in encouraging each other to continue taking the medication even if they experienced side effects.

*I think it is good because when a person is on this treatment, and they experience those side effects that we mentioned, one person may decide to default, but when we are as a group, we will encourage each other to endure.*

- FGD CARG member participant (with TPT experience)

Reduced burden on healthcare providers: The second widely perceived advantage was that integrating TPT into CARGs would reduce HCW workload and decongest HF.

*I think it is a good strategy because the workload at the clinic will be reduced, so if you train CARG leaders for them to train their CARGs, I think it will be good.*

- CARG leader key informant

Some participants also noted that the CARG leaders could play an active role in educating their members about TPT since HCW often do not have time to fully explain treatments to patients.

*I think there’s an advantage there because when the nurses [at the HF] give us our medication, they have limited time to teach us about things, so if the group leaders teach us instead, it will be helpful because they will take time to teach us.*

- FGD CARG member participant (with TPT experience)

Convenience: The participants emphasized that many PLHIV join CARGs to reduce the time and travel expenses associated with being on ART and that requiring frequent HF visits for TPT could become a barrier to uptake.

*People who have joined CARGs do so because of reasons of distance to the clinic or time, so a lot of people may not have time to go and spend a day at the clinic [to receive TPT].*

- Policy/program-level key informant

*It is good in that when a person does not have the time to visit the clinic, just like what we do with ARVs, we will do the same with distributing TPT.*

- CARG leader key informant

### 3.3. Perceived Challenges of Providing TPT through CARGs

The participants highlighted three potential disadvantages of integrating TPT into CARGs: medication stockouts due to multi-month dispensing (MMD) of TPT, decreased opportunities for HCW to monitor people on TPT, and concerns that the CARG leaders were insufficiently trained.

Medication stockouts: Although the participants appreciated the advantages of MMD for TPT and aligning the timing of TPT and ART pick-ups for people in CARGs, they expressed concerns that MMD could lead to TPT stockouts or create administrative challenges in terms of medication tracking and accountability.

*I think it will just create a logistics nightmare in terms of being able to account for the medicine....*

- Program/policy-level key informant

*A potential challenge is maybe coming here [the HF] and being told that there is no medication in stock ….*

- FGD CARG member participant (with TPT experience)

Limited HCW monitoring: A second concern was that the CARG leaders might be unable to monitor members for TPT side effects and/or symptoms of incident TB as effectively as HCW. Some program and policy-level stakeholders preferred that TPT be initiated at the HF, and others recommended models that included more frequent HF visits.

*…the patient needs to be assessed by a trained clinician… so that they can be able to carry on with their treatment without any problem … I would not recommend that.*

- Program/policy-level key informant

Insufficiently trained CARG leaders: When asked about their training, 16/20 (80%) of the CARG leaders reported receiving CARG-specific training. However, training on TB was lacking. While MoHCC guidelines require every CARG participant to be screened for TB symptoms at every meeting, 4/20 (20%) of CARG leaders noted screening for TB symptoms as one of their responsibilities. Study staff observed CARG leaders asking members if they currently had a cough in 2/8 meetings and if they had night sweats, weight loss, and/or contact with a person with active TB in 1/8 meetings. In contrast, ART adherence was assessed in 7/8 meetings. Participants stressed the importance of training CARG leaders on TB and TPT basics, TPT adherence support, TPT side effects, and the documentation of TPT services.

### 3.4. Perceived Advantages, Disadvantages and Preferences for TPT Delivery Models in CARGs

**Figure 2:**
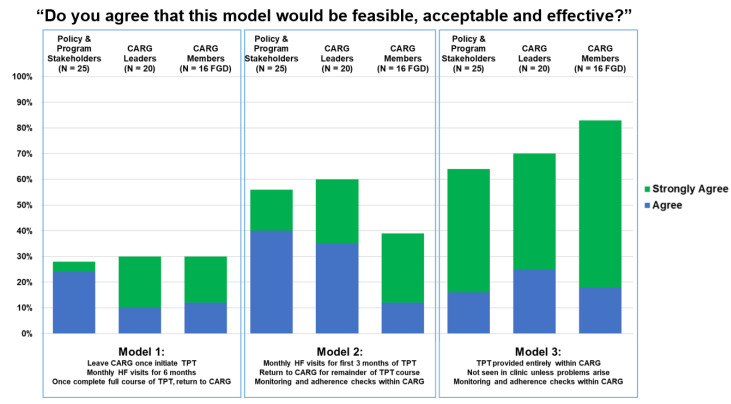
Participant reactions to hypothetical delivery models.

When presented with the three hypothetical TPT delivery models (Table 2), the participants indicated strong support for a model administered wholly within CARGs with multi-month TPT dispensing (Model 3). However, a sizeable number also agreed that Model 2 would be feasible, acceptable, and effective; this model included monthly HF visits for the first three months of TPT only (Figure 2). Table 4 illustrates the participants’ assessment of the advantages and disadvantages of each model.

## 4. Discussion

Zimbabwe is successfully scaling up TPT coverage for PLHIV but cannot reach its coverage goals by focusing solely on TPT at the time of ART initiation. In response, MoHCC is designing strategies that also target the 429,000 people established on ART and who are enrolled in less-intensive DSD models, recognizing that many of these individuals are eligible for TPT. In this study, we explored the perceived feasibility and acceptability of integrating TPT services into CARGs. We found that community-based TPT delivery for PLHIV in CARGs was highly acceptable to health system planners and policy makers, implementers, and CARG members living with HIV. A CARG-based approach aligning multi-month dispensing of TPT and ART with reduced frequency of HF visits was seen as more convenient, more efficient, and less disruptive for CARG members who could continue to receive services using a system that they trusted and that worked for them. However, some participants felt that it would be safer to initiate TPT at HF with monthly visits for the first three months.

The strengths of this study include its immediate policy relevance to expanding TPT coverage for PLHIV in Zimbabwe, its mixed-methods approach and robust sample sizes, and its inclusion of policy makers, implementers, and recipients of care to triangulate findings. The limitations of this include the relatively small number of HF sampled and the use of purposive samples for KII, FGD, and field observations, which limit generalizability.

Our findings align with experiences in other settings, in which policy makers, implementers, and PLHIV endorse the integration of TPT into community-based HIV models [9]. TPT has been piloted in DSD models in several countries. For example, TPT has been integrated into Zambia’s facility-based Fast Track model [1], South Africa’s facility-based club model [2], the community ART distribution point (points de distribution communautaires, PODI) model in the Democratic Republic of Congo [12], and into multiple DSD models in Uganda, including the community client-led ART delivery (CCLAD) model, which is similar to Zimbabwe’s CARG model [13]. One study in Uganda showed that PLHIV enrolled in a facility-based HCW-led visit spacing DSD model had higher TPT completion rates than PLHIV in standard ART delivery models [14].

Although study participants perceived community-based TPT delivery via CARGs to be both acceptable and feasible, they emphasized the importance of CARG leader training on TPT administration and side effects. This point was reinforced by the observation that CARG leaders did not adequately screen members for TB symptoms at CARG meetings, and that some had not received any training at all on how to manage CARGs. The importance of ongoing support for peer educators, peer supporters, and peer mentors has been highlighted in studies [15,16,17,18,19] and emphasized in implementation guidance for peer-led models [7,20]. Developing a competency-based training curriculum for CARG leaders and ensuring ongoing supportive supervision would be an important component of any future plans to deliver TPT in CARGs.

Participants were also concerned that MMD of TPT medications might lead to stockouts. This has not been a problem for Zimbabwe as it has shifted to the MMD of ART; its HIV treatment programs now routinely dispense three to six months’ worth of ART [21]. Global experience with MMD for ART has been largely positive; for example, MMD was found to be non-inferior to conventional treatment models in Zimbabwe [22], and studies of HCW in Zambia and Malawi found them to be strongly supportive of MMD for HIV treatment [23,24]. These experiences suggest that similar planning and stock management for TPT medication could avert this anticipated barrier. Zimbabwe is also shifting its TPT regimen from 6 months of daily isoniazid to three months of once-weekly isoniazid–rifapentine (3HP), which may also help to minimize the impact of MMD on supply chains.

## 5. Conclusions

Policy makers, implementers, funders, CARG leaders, and CARG members were confident that community-based TPT delivery could be integrated into CARGs. The key advantages of the integrated approach were felt to be its convenience, which the respondents felt would increase TPT uptake, the likelihood that adherence monitoring and support provided by peers and CARG leaders would be more effective than that provided by HCW, and efficiencies, including decreased burden on HCWs. Potential disadvantages included concerns that HCW might be more skilled than CARG leaders at monitoring for TPT side effects and that the multi-month dispensing of TPT might cause drug stockouts, and respondents recommended appropriate training and supportive supervision for CARG leaders and paying close attention to drug supply chains.

These findings complement the results of pilot studies in other countries and suggest that the integration of TPT service delivery into less-intensive DSD models, including CARGs, should be a priority for countries where TPT coverage amongst PLHIV is suboptimal. DSD models offer a platform with which to reach the cohort of people who are well established on ART but who may have missed out on access to TPT. By partnering with recipients of care and communities to design person-centered, contextually appropriate models for integrated TB/HIV care, Ministries of Health and implementers can scale up TPT to reach national and global targets and decrease TB-related morbidity and mortality.

## Figures and Tables

**Table 1:** Data collection strategy by sample and assessment domains.

Data Collection Strategy	Sample	Illustrative Domains
Key informant interviews with policy and program stakeholders (in person, 1 hour)	Policy and program stakeholders engaged in TPT services (e.g., policy makers, donors, and implementers)	- Familiarity with/understanding of TPT - Familiarity with DSD - Feasibility of providing TPT through CARGs - TPT implementation, monitoring and drug dispensing through CARGs - Needs for providing TPT through CARGs - Integration of other health services into CARGs
Key informant interviews with CARG leaders (in person, 1 hour)	CARG leaders at 7 health facilities	- Familiarity with/understanding of TPT - Experience as CARG leaders - Current CARG activities - Feasibility of providing TPT through CARGs - TPT implementation, monitoring, and drug dispensing through CARGs - Needs for providing TPT through CARGs - Integration of other health services into CARGs
Focus group discussions (in person, 90 min)	CARG members at 7 health facilities (6–10 participants per group)	- Familiarity with/ understanding of TB and TPT - Experience in CARGs - Feasibility of providing TPT through CARGs - TPT implementation, monitoring and drug dispensing through CARGs - Needs for providing TPT through CARGs - Integration of other health services into CARGs
CARG observations/time-motion study (30–75 min)	Observations of CARGs at the 7 health facilities using a structured observation checklist	- Key activities undertaken during CARG meeting: Individual health check-in Follow-up on health problems Support for new problems Adherence assessment Screening for TB symptoms - Documentation of activities - Amount of time spent on each activity - Challenges and successes

**Table 2:** Hypothetical delivery models.

Model 1	Once a person in a CARG initiates TPT, they leave the CARG model and are seen monthly at the clinic for the duration of TPT. They receive one month of TPT and ART at a time, with monthly clinical examinations. Once they complete the full course of TPT they return to the CARG model.
Model 2	Once a person in a CARG initiates TPT, they make monthly clinic visits for the first three months, and then return to the CARG for the remainder of the TPT course. Their fellow CARG members pick up three months’ worth of ART and TPT for them as usual, and their CARG leader monitors for adherence, side effects and symptoms of incident TB until they complete the full course of TPT.
Model 3	Once a person in a CARG initiates TPT, TPT is administered entirely within the CARG. In this model, the person on TPT is not seen in clinic after TPT initiation unless problems arise. They receive an initial three months of TPT and ART after which their fellow CARG members pick up three months’ worth of ART and TPT for them as usual, and their CARG leader monitors for adherence, side effects and symptoms of incident TB until they complete the full course of TPT.

**Table 3:** Characteristics of CARG members participating in FGDs.

		CARG Member FGD Participants	
		N = 136	
		**N**	**%**
Age	Median	46	
	Range	20–66	
	IQR	42–54	
Sex	Female	92	68%
	Male	44	32%
Years on ART *	Median	8	
	Range	1–19	
	IQR	6–11	
Years as CARG member	<1	10	7%
	1–2	58	43%
	>2	68	50%
Household or family member in your CARG	None	69	51%
	One—partner or spouse	47	35%
	One—child	5	4%
	One—other	10	7%
	More than one	5	4%
Marital status	Single (never married)	4	3%
	Married or cohabiting	79	58%
	Divorced or separated	18	13%
	Widowed	35	26%
Highest level of education	None	6	4%
	Some primary	44	32%
	Some secondary	76	56%
	Some tertiary or higher	10	7%
Income earned last month	≤USD 100	78	57%
	USD 101–USD 500	29	21%
	USD 501–USD 1000	4	3%
	≥USD 1000	1	1%
	Don’t know/no answer	24	18%

* N = 135.

**Table 4:** Participants’ assessment of advantages and disadvantages of the three hypothetical models.

		Model 1	Model 2	Model 3
Perceived Advantages	More-intensive in-person monitoring by HCW may increase client safety	X *	X	
	Documentation of client monitoring and TPT dispensing is easier for HCW if client is at health facility	X	X	
	Monthly dispensing may reduce TPT stock-outs	X	X	
	Integrated TPT/ART adherence monitoring and support from CARG leaders and peers may be more effective than monitoring and support provided by HCW		X	X
	The convenience of an integrated model may increase TPT uptake		X	X
Perceived disadvantages	More frequent visits increase HCW workload	X	X	
	The inconvenience of more frequent HF visits may decrease client willingness to take TPT	X	X	
	Having to leave the support of a CARG may decrease ART adherence	X	X	

* X indicates that the issue was identified as an advantage or disadvantage of the hypothetical model by KII and/or FGD participants.

## Data Availability

The first author will provide all data supporting reported results upon request.

## Abbreviations

The following abbreviations are used in this manuscript:

3HP	Three months of once-weekly isoniazid-rifapentine
ART	Antiretroviral therapy
CARG	Community ART Refill Groups
CCLAD	Community client-led ART delivery
DSD	Differentiated service delivery
FGD	Focus group discussion
HCW	Health care workers
HF	Health facility
HHS	The U.S. Department of Health and Human Services
HIV	Human immunodeficiency virus
HRSA	Health Resources and Services Administration
ICAP	ICAP at Columbia University
IQR	Interquartile range
KII	Key informant interview
MMD	Multi-month dispensing
MOHCC	Ministry of Health and Child Care
PLHIV	People living with HIV
PODI	Points de distribution communautaires
TB	Tuberculosis
TPT	Tuberculosis preventive treatment
ZNNP+	Zimbabwe National Network of People Living with HIV
